# A Comparison on LSTM Deep Learning Method and Random Walk Model Used on Financial and Medical Applications: An Example in COVID-19 Development Prediction

**DOI:** 10.1155/2022/4383245

**Published:** 2022-08-23

**Authors:** Yifan Yao, Xinxin Li, Qing Li

**Affiliations:** ^1^School of Fintech, Hebei Finance University, Baoding, China; ^2^Finance Department, Capital University of Economics and Business, Beijing, China

## Abstract

This study aims to establish the model of the cryptocurrency price trend based on a financial theory using the Long Short-Term Memory (LSTM) networks model with multiple combinations between the window length and the predicting horizons. The Random Walk model is also applied with different parameter settings. The object of this study is the cryptocurrency and medical issues, primarily the Bitcoin and Ethereum and the COVID-19. Quantitative analysis is adopted as the method of this dissertation. The research tool is Python programming language, and the TensorFlow package is employed to model and analyze research topics. The results of this study show the limitations of the LSTM and Random Walk model for price prediction while demonstrating the different characteristics of both models with different parameter settings, providing a balance between the model's accuracy and the model's practicality.

## 1. Introduction

The subject of general dynamics for digital currencies is a popular one in the literature of modern cryptocurrency analysis [1]. In 2017, the volume of cryptocurrency transactions increased dramatically due to the capital market's ultraexponential growth [2]. However, the movement of cryptocurrency exhibits high volatility, adding more uncertainty to the transaction market. Most articles on cryptocurrency and machine learning focus on the problems of model prediction [3, 4]. However, many ignore the mathematical principles behind the model regardless of the relationship between accuracy and parameter settings. This leads to some seemingly accurate models that are not generally practical. This article will explore the relationship between mathematical principles and model accuracy and discuss the essence through phenomena. Considering that there are two theories in the financial market, one is that the stock price is predictable [5], and the other is that the stock price is entirely unpredictable [6], which indicates that the price is a Random Walk, the machine learning model described below (e.g., LSTM (Long Short-Term Memory networks) and RNN (recurrent neural network)) will verify the predictable hypotheses, and Random Walk theory is also applied in this article, which will be researched based on the previous study [7–9]. With the global epidemic outbreaks, financial development is primarily affected by COVID-19. The motivation of the study is to explore the machine learning model's performance in both contexts and find an optimal potential parameter combination to explain the unstable trends and some common ones. The study will first experiment with the mentioned financial problem and then apply the result in the COVID-19 model prediction case to verify the model parameters in different contexts and conclude an optimal parameter settings combination.

The Long Short-Term Memory (LSTM) and recurrent neural network (RNN) models are frequently applied in this field, which are preferred over the conventional multilayer perceptron [10]. Sean McNally compared the RNN and the LSTM model used on Bitcoin [11], for the RNN (recurrent neural network) implementation part. The author first took the temporal length window by the autocorrelation function. In the LSTM part, the previous research [12] has illustrated that, compared with the RNN, the LSTM outperforms RNN and ARIMA at learning long-term dependencies. The ARIMA (Autoregressive Integrated Moving Average) model is a time series model often used in the price prediction [13, 14]. A model comparison is presented [12] in Table 1.

The Long Short-Term Memory (LSTM) and recurrent neural network (RNN) are frequently applied in this field [10]. Table 1 shows that the precision and accuracy do not significantly differ between the two models. Both LSTM and RNN models are capable of training data with LSTM being more applicable to the long-term dependencies.

As for the multiple window length settings [15], different window sizes are applied based on the LSTM model to capture better features of the equipment, which concluded that various time window sizes have a positive impact on recognizing various temporal dependencies among features, while [16] used ten combinations of sliding windows with prediction ranges to explore the accuracy improvement possibility for deep learning fully and concluded that if the window length is small while the prediction range is far ahead simultaneously,the RMSE (Root Mean Square Error) will become lower than the primary method.

A nonlinear model should be applied to this topic in accordance with volatility. Many scholars have compared the RNN (recurrent neural network) and the LSTM (Long Short-Term Memory). According to the results, the LSTM model outperforms RNN, since it is more suitable for long dependencies. Significantly, the window sliding method with the different prediction range variables should be applied in this article. Furthermore, the theory of Random Walks in cryptocurrency prices was also experimented with respect to the predictable price hypothesis.

To sum up, the structure of this article is divided into seven parts: introduction, literature review, methodology, data collection, implementation, discussion, and conclusion and future work. The details are shown in Table 2.

## 2. Literature Review of Related Work

As people's health awareness and philosophy increase, how to more effectively improve the utilization of medical resources has become an issue of concern to society at large. Some literature on medical applications pays particular attention to the wireless sensor network (WSN) technology, a spatially distributed sensor node that aims for important information collection [17]. For example, the study [17] proposed a multiagent-based architecture for WSNs and particle swarm optimization (PSO) algorithm to improve the model ability of the population diversity issue. Besides, based on Ant Colony Optimization (ACO), the study [18] also proposed a novel adaptive intelligent routing scheme for WSNs to achieve a better model performance in terms of energy consumption and efficiency. Moreover, an energy-efficient sleep scheduling mechanism (ESSM) is also proposed for WSNs to reduce energy consumption effectively [19]. Apart from WSN technology application, deep learning methods and biometric methods are also carried out in some medical issues. For example, the study in [20] used a biometric method, which is a finger vein personal authentication method, and the study in [21] used a deep learning method of XGBoost and genetic algorithm to extract pedestrian feature which is an inspiration of object recognition in medical problems.

## 3. Methodology

### 3.1. Principle and Introduction of LSTM Model

#### 3.1.1. Start from RNN

**Figure 1:**
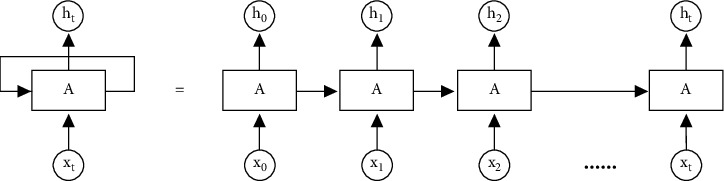
The structure of RNN.

RNN represents the recurrent neural network, and time is a significant impact factor for RNN [22]. The output comes out with each moment's input combined with the state of the current model. In Figure 1, the output h_t_ comes out with both the input *xt* and the hidden state from moment *t*−1, which is provided by the looped edge. Theoretically, the recurrent neural network can be capable of sequences of arbitrary length [23]. However, in practice, the problem of gradient dissipation or explosion will happen during the optimization for the too long sequence. Furthermore, the dissipation of the gradient will make the weight of previous layer not updated during the forward propagation; on the contrary, the gradient explosion will make training process unstable; thus, the model cannot obtain the optimal parameters.

#### 3.1.2. Mathematical Explanation of RNN

**Figure 2:**
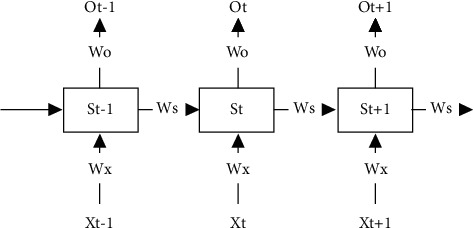
The inner structure of RNN.

Given the 3 moments of RNN unit, in Figure 2, assuming that the left input *S*_0_ is a given value and no activation function exists in the neuron, subsequently, the forward process is expressed as

(1) $$\begin{array}{c} S_{1}=W_{x}X_{1}+W_{s}S_{0}+b_{1}O_{1}=W_{o}S_{1}+b_{2},\\ S_{2}=W_{x}X_{2}+W_{s}S_{1}+b_{1}O_{2}=W_{o}S_{2}+b_{2},\\ S_{3}=W_{x}X_{3}+W_{s}S_{2}+b_{1}O_{3}=W_{o}S_{3}+b_{2}. \end{array}$$

At the time of *t* = 3, the loss function can be written as

(2) $$\begin{array}{c} L_{3}=\frac{1}{2}{\left(Y_{3}-O_{3}\right)}^{2}. \end{array}$$

RNN training is virtually to seek partial derivatives of *W*_0_, *W*_*x*_, *W*_*s*_, *b*_1_, *b*_2_, adjusting them in order to obtain the minimum of *L*_3_. According to the chain rule,

(3) $$\begin{array}{c} \frac{\delta L_{3}}{\delta W_{0}}=\frac{\delta L_{3}}{\delta O_{3}}\frac{\delta O_{3}}{\delta W_{0}},\\ \frac{\delta L_{3}}{\delta W_{x}}=\frac{\delta L_{3}}{\delta O_{3}}\frac{\delta O_{3}}{\delta S_{3}}\frac{\delta S_{3}}{\delta W_{x}}+\frac{\delta L_{3}}{\delta O_{3}}\frac{\delta O_{3}}{\delta S_{3}}\frac{\delta S_{3}}{\delta S_{2}}\frac{\delta S_{2}}{\delta W_{x}}+\frac{\delta L_{3}}{\delta O_{3}}\frac{\delta O_{3}}{\delta S_{3}}\frac{\delta S_{3}}{\delta S_{2}}\frac{\delta S_{2}}{\delta S_{1}}\frac{\delta S_{1}}{\delta W_{x}},\\ \frac{\delta L_{3}}{\delta W_{x}}=\frac{\delta L_{3}}{\delta O_{3}}\frac{\delta O_{3}}{\delta S_{3}}\frac{\delta S_{3}}{\delta W_{s}}+\frac{\delta L_{3}}{\delta O_{3}}\frac{\delta O_{3}}{\delta S_{3}}\frac{\delta S_{3}}{\delta S_{2}}\frac{\delta S_{2}}{\delta W_{s}}+\frac{\delta L_{3}}{\delta O_{3}}\frac{\delta O_{3}}{\delta S_{3}}\frac{\delta S_{3}}{\delta S_{2}}\frac{\delta S_{2}}{\delta S_{1}}\frac{\delta S_{1}}{\delta W_{s}}. \end{array}$$

It is briefed as 

(4) $$\begin{array}{c} \frac{\delta L_{3}}{\delta W_{x}}=\sum_{k=0}^{t}\frac{\delta L_{t}}{\delta O_{t}}\left(\prod_{j=k+1}^{t}\frac{\delta S_{j}}{\delta S_{j-1}}\right)\frac{\delta S_{k}}{\delta W_{x}},\\ \frac{\delta L_{3}}{\delta W_{3}}=\sum_{k=0}^{t}\frac{\delta L_{t}}{\delta O_{t}}\frac{\delta O_{t}}{\delta S_{t}}\left(\prod_{j=k+1}^{t}\frac{\delta S_{j}}{\delta S_{j-1}}\right)\frac{\delta S_{k}}{\delta W_{s}}. \end{array}$$

This formula suggests that the ∏_*j*=*k*+1_^*t*^*δS*_*j*_/*δS*_*j*−1_ part causes the gradient dissipation or explosion. With the activation function added, it is expressed as

(5) $$\begin{array}{c} S_{j}=\text{tanh}\left(W_{x}X_{j\;}+W_{x}S_{j-1}+b_{1}\right), \end{array}$$

and it is concluded that

(6) $$\begin{array}{c} \overset{t}{\underset{j=k+1}{\prod }}\frac{\delta S_{j}}{\delta S_{j-1}}=\prod_{j=k+1}^{t}\tanh^{\prime }W_{s}, \end{array}$$

where tanh derivative is always below 1. With the increase in *t*, the above formula's value turns closer to zero as long as *W*_*s*_ is above 0 and below 1 as well, leading to the disappearance of the gradient. Subsequently, the above formula will become more and more infinite if *W*_*s*_ is large, thus producing a gradient explosion, which explains why the LSTM is introduced.

#### 3.1.3. Mathematical Explanation of LSTM Model

LSTM represents the Long Short-Term Memory, an RNN type. *C*_*t*_ is called current cell state, which can be expressed as

(7) $$\begin{array}{c} c_{t}=f_{t}⊕c_{t-1}+i_{t}⊕\text{tanh}\left(W_{c}\left[h_{t-1\;},x_{t}\right]+b_{c}\right), \end{array}$$

and *f*_*t*_ is called the forget gate, which can be expressed as

(8) $$\begin{array}{c} f_{t}=\sigma \left(W_{f}\left[h_{t-1\;},x_{t}\right]+b_{f}\right), \end{array}$$

deciding which features can be employed for the calculation of *C*_*t*_ from *C*_*t*−1_. The current hidden output can be expressed as

(9) $$\begin{array}{c} h_{t}=o_{t}⊗\text{tanh}\left(c_{t}\right). \end{array}$$

Besides, the input and output gates are expressed, respectively, as

(10) $$\begin{array}{c} i_{t}=\sigma \left(W_{i}\left[h_{t-1\;},x_{t}\right]+b_{i}\right)\\ o_{t}=\sigma \left(W_{0}\left[h_{t-1\;},x_{t}\right]+b_{o}\right). \end{array}$$

**Figure 3:**
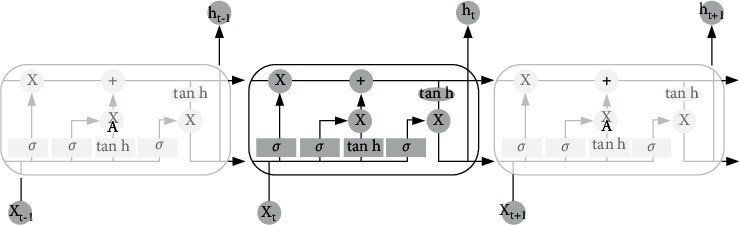
The structure of LSTM.

The above formulas show that the activation function of 3 gates is sigmoid, revealing that the output of these three gates is either close to 0 or close to 1. This makes *δc*_*t*_/*δc*_*t*−1_ = *f*_*t*_ , *δh*_*t*_/*δh*_*t*−1_ = *o*_*t*_ part become 0 or 1. When it is 1, the gradient can be transmitted well in the LSTM, significantly reducing the probability of the gradient dissipation. When the gate is 0, the information at the previous moment does not impact the current moment, indicating that there are no instructions to transmit the gradient backwards for updating the parameters [24]. Accordingly, this explains the reason why the gradient can be solved using the LSTM model shows in Figure 3.

#### 3.1.4. Mathematical Explanation of Random Walk Model

For the time series {*x*_*t*_}, if it satisfies *x*_*t*_=*x*_*t*−1_+ *w*_*t*_, where *w*_*t*_ denotes a white noise with a mean of 0 and a variance of *σ*^2^, the sequence {*x*_*t*_} will be a Random Walk [37]. By definition, *t* at any *x*_*t*_  moment refers to the sum of all historical white noise sequences that do not exceed the *t* moment, so it is concluded that

(11) $$\begin{array}{c} x_{t}=w_{t}+w_{t-1}+w_{t-2}+\cdots +w_{0}. \end{array}$$

The sequence mean and variance of Random Walk are presented as follows:

(12) $$\begin{array}{c} \mu_{x_{t}}=0,\\ \text{var}\left(x_{t}\right)=\text{var}\left(w_{t}\right)+\text{var}\left(w_{t-1}\right)+\;\cdots \text{var}\left(w_{0}\right)\;\\ =t\times \text{var}\left(w_{t}\right)\\ =t\sigma^{2}. \end{array}$$

Although the mean does not change with time *t*, due to the fact that the variance is the function that relates to *t*, the Random Walk does not satisfy the stability. As time *t* and the variance of *x*_*t*_ are regulated, the stability is upregulated. For the given interval *k*, the Random Walk covariance is performed as

(13) $$\begin{array}{c} \text{Cov}\left(x_{t}\;,x_{t+k}\right)=\text{Cov}\left(x_{t}\;,x_{t}+w_{t+1\;}+\cdots +w_{k}\right)\\ =\text{Cov}\left(x_{t}\;,x_{t}\;+\sum_{i\;=\;t+1}^{k}\text{Cov}\left(x_{t},w_{i}\right)\;\right)\\ \text{Cov}=\left(x_{t}\;,x_{t}\right)+0\\ =t\sigma^{2}. \end{array}$$

From the concluded variance and covariance, the autocorrelation function *ρ*_*k*_ (*t*) is calculated as follows:

(14) $$\begin{array}{c} \rho_{k}\left(t\right)\frac{\text{Cov}\left(x_{t},x_{t+k}\right)}{\sqrt{\text{Var}\left(x_{t}\right)}\sqrt{\text{Var}\left(x_{t+k}\right)}}\\ =\frac{t\sigma^{2}}{\sqrt{t\sigma^{2}}\sqrt{\left(t+k\right)\sigma^{2}}}\\ =\frac{1}{\sqrt{1+k/t}}. \end{array}$$

Clearly, the autocorrelation function is related to time *t* and interval *k*, indicating that if the Random Walk model has a long time series while the interval is quite small, the autocorrelation coefficient is approximated as 1. In other words, if there is a model predicting the stock price based on time *t* as the forecast for the *t*+1 value, the correlation coefficient between the actual value and the predicted value equals the stock price sequence of *k*=1. In other words, the forecast of today's price as tomorrow's price is also very close to 1, which will mislead us into thinking that the model is accurate.

## 4. Data Collection

The financial data are all collected from the CoinMarketCap, which is an authoritative website committed to cryptocurrency market value statistics. Only the Bitcoin and Ethereum data are adopted to train the LSTM model and the Random Walk model. The raw ranges from April 2017 to December 2020 for nearly 3 years span. The training size parameter is 0.8, while the test size reaches 0.2. Meanwhile, the COVID-19 cases data is obtained in National Statistical Office, ranging from March 2020 to July 2020 in China; given that the mentioned period witnessed the peak of the global epidemic, it might be representative.

## 5. Implementation

### 5.1. Training Process of Random Walk Model

#### 5.1.1. Single-Point Method Prediction

**Figure 4:**
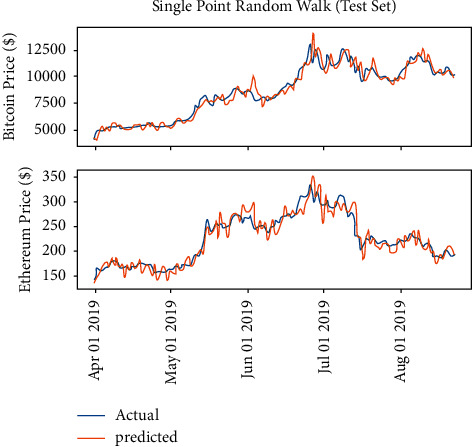
Singe point Random Walk model performance.

From the preliminaries illustrated below, the Random Walk model will learn parameter *σ*, which is the only parameter of the Random Walk.  Figure 4 shows the model performance.

**Figure 5:**
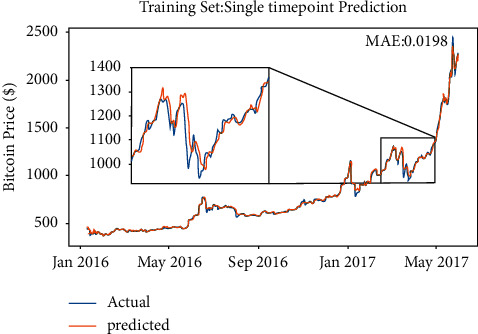
The details of single-point prediction on Bitcoin.

**Figure 6:**
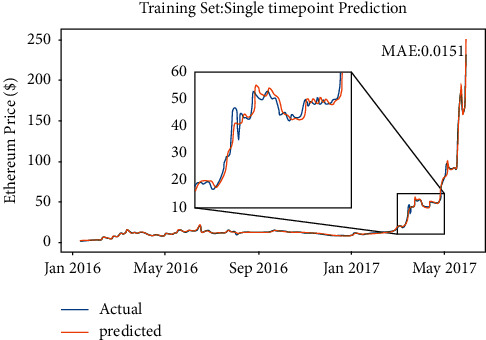
The details of single-point prediction on Ethereum.

Based on the preliminaries, the single-point Random Walk model seems to be performing well, which is in accordance with expectation. The model just predicts the next day, so *k*=1. Besides, the time span is 3 years, suggesting that *t* is very large, so *ρ*, *k*=1, implying that the forecast of next day is just the repeat of the current day, and, due to the single-point method selection, the error will reset every time, which means that every next input will be the true data. Figures 5 and 6 suggest that the prediction line is similar to the copy in the horizontal direction. The model seeming accurate is attributed to the mathematical nature of Random Walk rather than the training process. Here, the model trained by the data in 2017 shows the details of the copy in the horizontal direction.

#### 5.1.2. Multipoint Method Prediction

**Figure 7:**
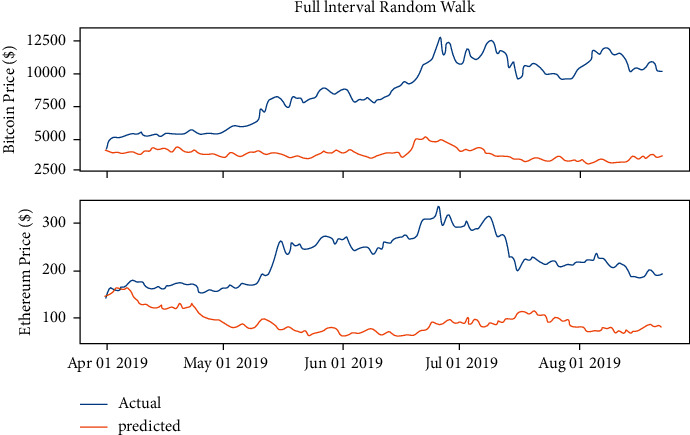
Full interval Random Walk performance.

As mentioned below, if the model intends to ignore the misleading accuracy caused by the nature of Random Walk [25], increasing the value of *k* can solve this problem. That is to say, the interval of the Random Walk step will be larger instead of +1 days. Therefore, a multipoint prediction method is proposed. In such way, the error cannot be reset, which will be exacerbated by subsequent predictions. The training result can be seen in Figure 7.

Obviously, changing the value of *k* will cause a significant reduction in the model accuracy; *p*_*k*_(*t*) will not approach 1 with the increase of *k*. That is to say, the result of the model is not associated with the nature of the Random Walk model. What is more, because the errors will be compounded by subsequent predictions, the predicting line is penalized seriously. What needs to be noticed is the fact that the Random Walk model is defined as *x*_*t*_= *x*_*t*−1 _+*w*_*t*_. That is, the price of the day is randomly changed based on the price of the previous day, while the price difference is all included in the random item *w*_*t*_. It can be seen from the above Random Walk model that the time series of the securities price will be in a random state and will not exhibit a certain observable or statistically determined trend. Compared with the machine learning model, the Random Walk model only explores the random item *w*_*t*_; it does not learn from the inputs or learn any parameters or weights of the model. That is why the single-point model or the multipoint model are both not the ideal solution for predicting the trend of cryptocurrency.

### 5.2. Training Process of LSTM Model

#### 5.2.1. Point-to-Point Method Prediction

**Figure 8:**
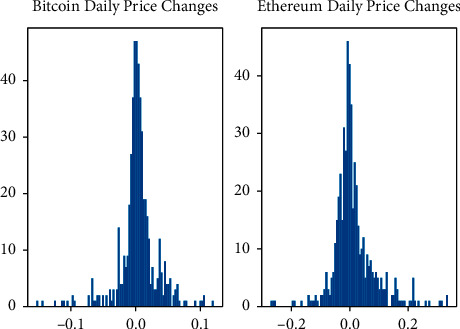
Daily price changes on Bitcoin and Ethereum.

The LSTM created is a two-dimensional model using only the close price and the transaction volume features, considering the price of changes daily is an immense difference every period as Figure 8 shows, which means that the model will not converge, so the normalizing operation [26] might be required.

For the training data, to normalize the price changes, equation (15) is used, where *p*_*i*_ represents the current window price and *p*_0_ is the next window price. So the input and output will be a percentage format. For the test data, the output will be denormalized as a direct real price of prediction is expected to visualize; for the denormalization, equation (16) will be used.

(15) $$\begin{array}{c} n_{i}=\left(\frac{p_{i}}{p_{0}}-1\right), \end{array}$$

(16) $$\begin{array}{c} p_{i}\;=p_{0}\left(n_{i}+1\right). \end{array}$$

Here the model uses MAE (Mean Absolute Error) [27] equation to validate the error between the predicted value and the true value, which is the average of absolute errors that can better reflect the actual situation of the prediction value error.

**Figure 9:**
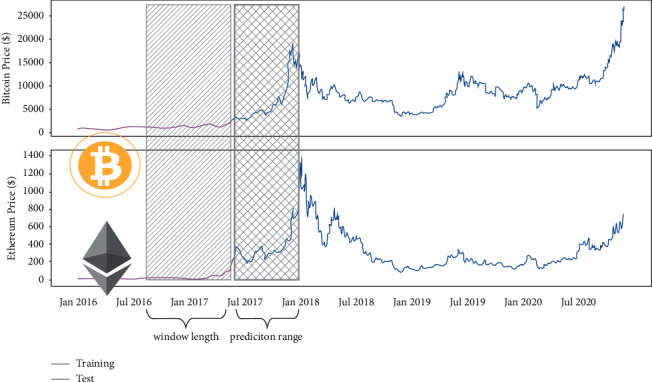
The explanation of window length and prediction range.

After the selection of parameters, the training dataset is used to train the model. The merge date starts from 2017 to 2020 and the split size is 0.8, so the training dataset is mainly from 05 in 2017 to 10 in 2019.  Table 3 shows the Bitcoin training process of the model; it is obvious that ,from epoch 18, the model started to converge as it lastly nearly stays at the MAE of 0.0330.  Figure 9 shows the LSTM training process of the Bitcoin.

**Figure 10:**
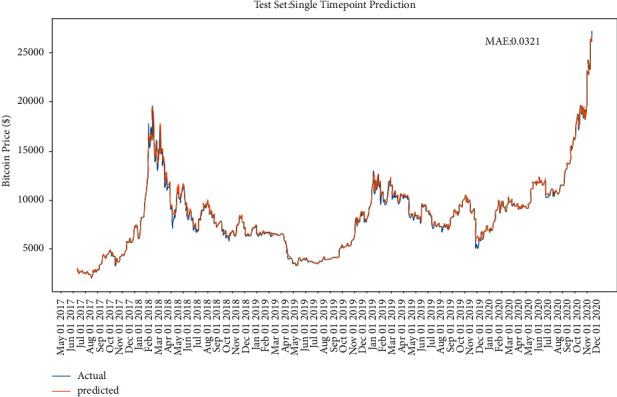
The performance of LSTM with single-point prediction on Bitcoin.

**Figure 11:**
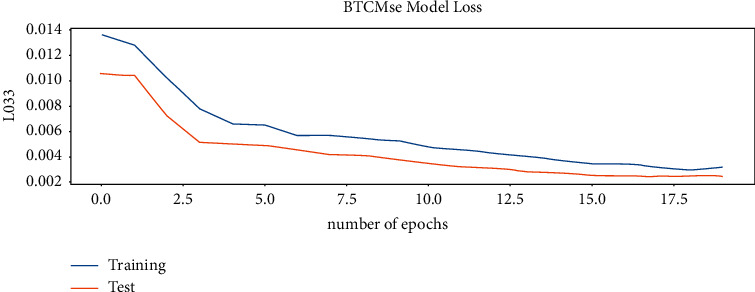
The model loss on Bitcoin.

**Figure 12:**
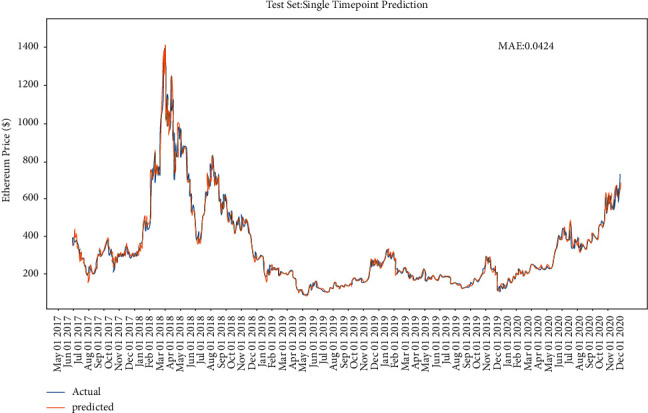
The performance of LSTM with single-point prediction on Ethereum.

**Figure 13:**
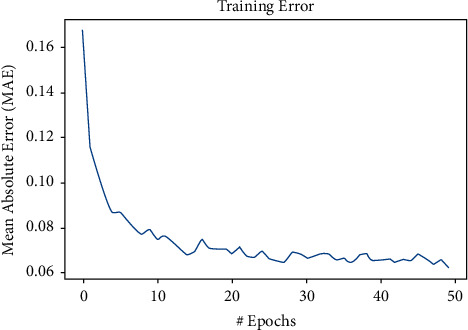
The training error on Ethereum.

After the convergence of the model [28], it is applied to the test dataset, which ranges from 11 in 2019 to 12 in 2020. The performance of the model on the Bitcoin test dataset is shown in Figure 10. As in Figure 11, both training set and the test set stop decreasing at epoch 20; after epoch 20, the training set error will still decrease, but the error on the test set will start to increase due to the model overfitting problem. Figures 12 and 13 show the performance of the model on Ethereum.

**Figure 14:**
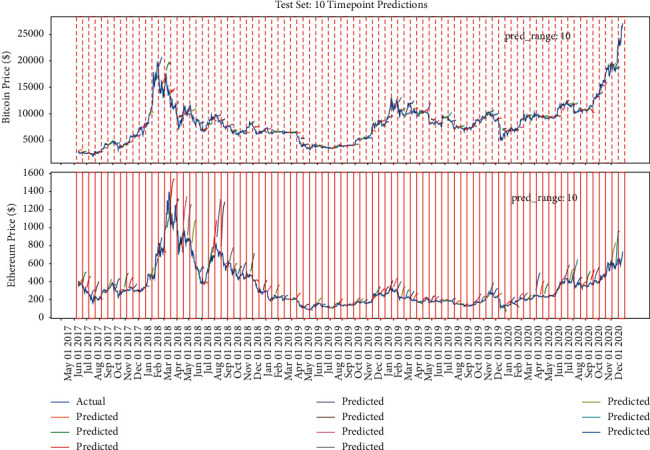
The model's performance with window length = 10 and prediction range = 10.

The model in this part used the point-to-point method. The point-to-point prediction is the process of making the model predict one single-point value each time and plot the corresponding position in the figure; after predicting this point, the window will slide to next point with the complete test data. Besides, the point-to-point method seems to be more accurate than the full interval prediction [29], whereas it does not imply that the point-to-point model outperforms the full interval model, since the error generated by each single prediction is reset each time, the neural network itself does not need to know the time series itself, and all the inputs are based on the real value in every next prediction. For the ignorance of the errors, the model seems unsurprisingly accurate. Furthermore, in Figure 14, it is suggested that the predicted value is more like a horizontal translation of the true value. For instance, from mid-May to mid-June 2019, several prices increased, and the peaks were following the fluctuations of the true values, which has an obvious hysteresis. In other words, the deep learning LSTM model regenerates an autoregressive model of order *p*; in these datasets area, the predicted value is the weighted sum of the previous *p* values, as defined below:

(17) $$\begin{array}{c} \text{PredPrice}=w_{0}+\;w_{1}*{\text{Price}}_{t-1}+\cdots w_{p}*{\text{Price}}_{t-p}+\epsilon_{t},\epsilon_{t}∼N\left(0,\sigma \right), \end{array}$$

where the next prediction will only be the true Price_*t-p*_ value with the calculated weight because the point-to-point method will ignore the error of every previous prediction, which largely reduces the inaccuracy. Therefore, in order to maximize the advantages of LSTM based on time series and avoid the model updating the error at every step, the model will be improved from the two following indicators: the first is window length, and the second is the prediction range. Window length is the historical time used by LSTM, and the prediction range refers to the range of backward prediction by the data trained in window length (Figure 9).

#### 5.2.2. Multipoint Method Prediction with Fixed Window Length Selection

Unlike the limitation of point-to-point training method, the multiple time-point method is more practical. Likewise, it initializes the test window and keeps moving to predict next point [30]. Besides, it will move forward a full window size and resets the window with true test data, while it moves to the point where the input window is already constituted by full past *Xt*−1 predictions. Thus, during the prediction, the error will not be fully reset, whereas the error will be accumulated in each full predicted window length, and the error will be reset again in a new window length. The error subtraction is shown in Table 4. For this reason, the model will be more practical. It is neither as deceptive as single-point prediction nor does it completely detour the model from the trajectory of the real point.

**Figure 15:**
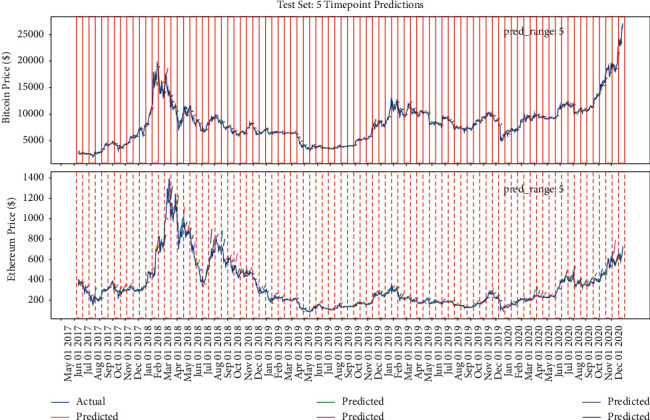
The model's performance with window length = 10 and prediction range = 5.

**Figure 16:**
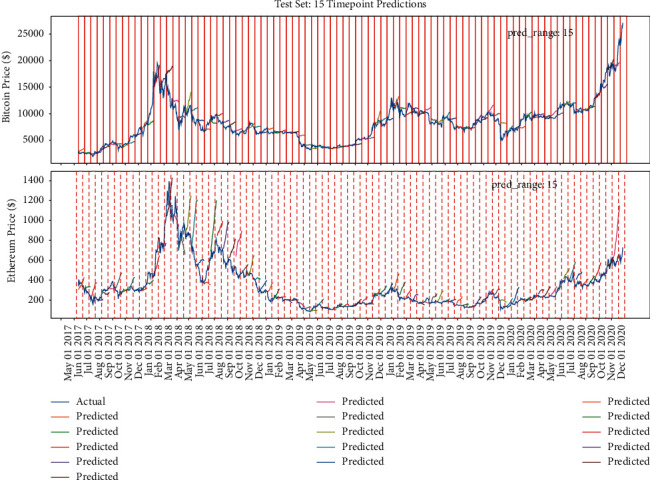
The model's performance with window length = 10 and prediction range = 15.

**Figure 17:**
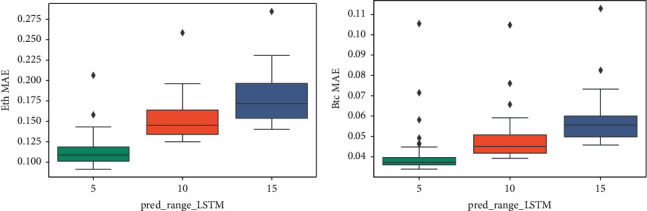
The box-plot of MAE based on different prediction ranges. (a) Ethereum MAE. (b) Bitcoin MAE.

Figures 15, 14, and 16 show that the multiple sequence LSTM does not perform well as expected. Besides, the red line in the figure is the prediction range. In the training process, the prediction range is set, respectively, at [5, 10, 15], while the window length is set at 10. The prediction of the model in each range does not reflect the price of the next trend, and the model seems to only predict the upward trend of the trend, while the price decline trend model does not seem to be aware. This may be due to the selection of parameters or the selection of the length of the window, which reduces the model accuracy. In addition, Figure 17 points out that when the window length is fixed, as the number of prediction points increases, the MAE increases accordingly, which indicates that, in the condition of the same window length selection, the model will be more accurate with less number of points.

#### 5.2.3. Fixed Multipoint Method Prediction with Different Window Length Selection

**Figure 18:**
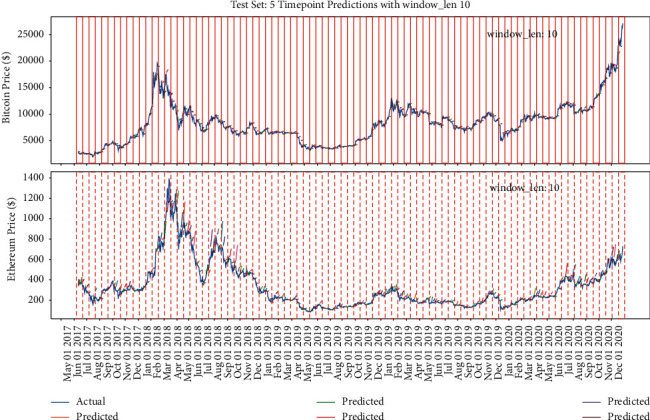
The model's performance with window length = 10 and prediction range = 5.

**Figure 19:**
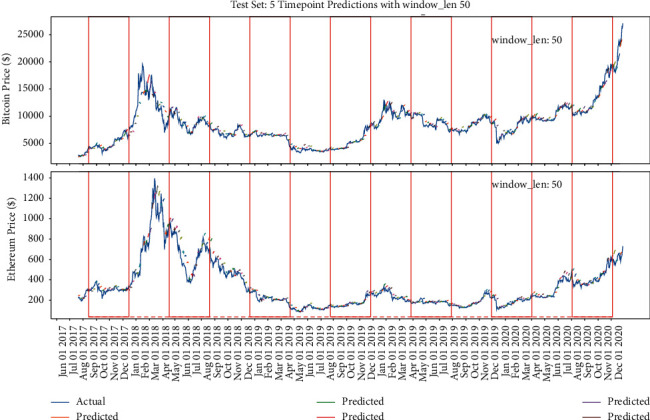
The model's performance with window length = 50 and prediction range = 5.

**Figure 20:**
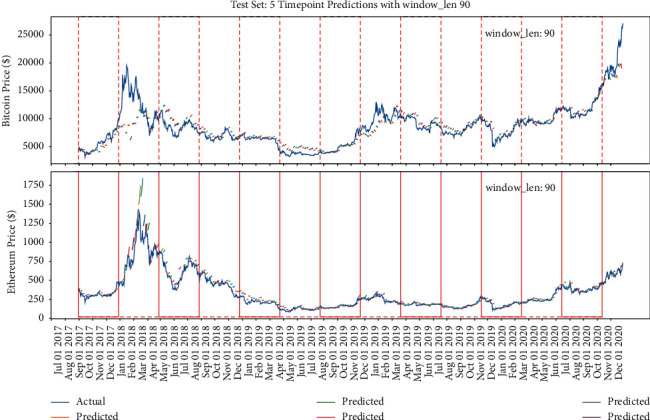
The model's performance with window length = 90 and prediction range = 5.

The previous part verifies the impact of different amount of points selection on the model when the window length is fixed. This part will verify the impact of different window length on the accuracy of the model when the amount of points selection is fixed. Similarly, the red lines in Figures 18–20 refer to the different window length settings. In the training process, the window length is set, respectively, at 10, 50, and 90, while the prediction range is set at 5.  Figure 18 shows that, at the condition of window length = 10, the model prediction trend performs similarly to the previous part, which seems to only predict the upward trend regardless of the decrease trend, while when the window length = 50, the model could reflect the correct decrease trend generally, and when the window length = 90, the model could reflect all the trend but is not basically right; especially during the period from May 2019 to August 2019, the decrease trend of Bitcoin prediction is totally wrong.

**Figure 21:**
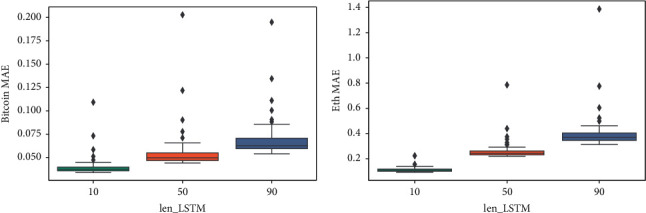
The box-plot of MAE based on different window length. (a) Bitcoin MAE. (b) Ethereum MAE.

From Figure 21, it could be concluded that, with the fixed prediction range, the model accuracy decreases with the increase of the window length, which is caused by the accumulation of errors [31] in the model.

#### 5.2.4. Exploration of LSTM Performance on COVID-19

**Figure 22:**
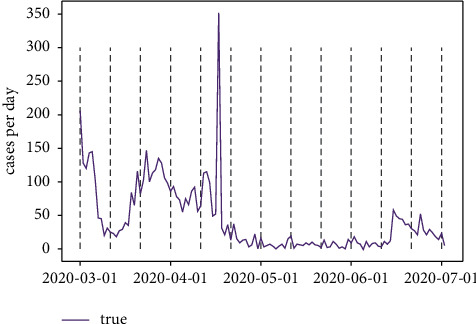
COVID-19 trend in 2020 (March to June).

According to the illustration of prediction range and window length based on the LSTM regarding the cryptocurrency problems, the parameters are concluded as 5 and 10, respectively. In order to test if other circumstances also satisfy this parameter combination, a COVID-19 growth cases per day prediction is introduced. It could be seen that the epidemic trend decreased significantly from the start of March to mid-March and increased back to 150 cases per day. The diagnosed cases reached the peak and decreased dramatically in mid-April and gradually maintained a flattening trend.  Figure 22 shows the trends.

**Figure 23:**
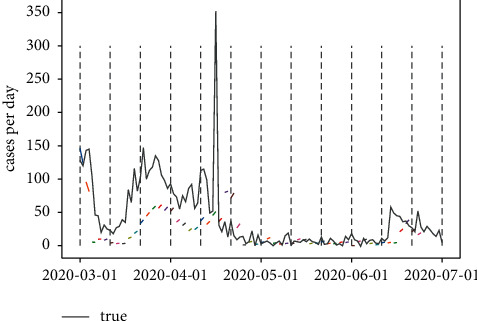
Window length = 10; prediction range = 1.

**Figure 24:**
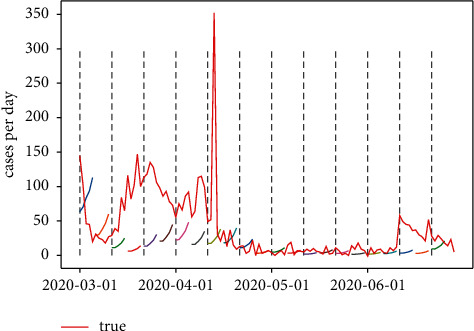
Window length = 10; prediction range = 5.

**Figure 25:**
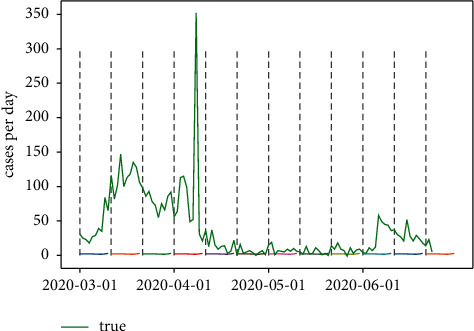
Window length = 10; prediction range = 10.

In order to explore the different parameter settings effect based on the COVID-19 data, three combinations of window length and prediction range are applied, and the results are shown in Figures 23–25. The grey-dotted lines in the following figures are the certain window length of 10, whereas the short lines represent the different prediction range.

From Table 5, it is clear that the model prediction effect was the same at the parameter's combination of 10 and 5, which is in line with the financial problems mentioned previously. The loss error of the LSTM model reached minimum at 0.0015; what should be noticed is the fact that the peak time during mid-April was not reflected by the model; given that the window length is 10, the model is limited by its deferral nature. In most cases, however, the model can exhibit some degree of slowdown in the rate of growth, which might be suggestive for the medical officials.

## 6. Discussion

According to Tables 3, 5, and 6, after using different combinations of window length and prediction range, it is found that when window length = 10 and prediction range = 5, the Bitcoin and Ethereum LSTM models reach minor errors, which are 0.037 and 0.113, respectively; in the circumstances of predicting the COVID-19 case, the model applied the same parameters combinations in line with the financial problem. It turned out that the parameters window length = 10 and prediction range = 5 are also optimal. In this regard, the combination of window length = 10 and prediction range = 5 should be suggested in future work. Besides, it can be seen that although the single-point method has the smallest error, it is the result of the error being reset every time. However, in the real financial price market, only predicting the price trend of the next day is impractical. Thus, predicting the price over a period of time with a proper error reset frequency is more practical, that is, to have a specific prediction range.

Table 7 shows the relationship between the interval of days and the accuracy. It can be seen that the error is not as significant as expected. Therefore, it can be concluded that, with a particular model accuracy guaranteed, the model has the best balance of practicality and accuracy based on the combination of window length = 10 and prediction range = 5. In summary, it can be concluded from the study of the Random Walk model and LSTM model that it is not appropriate to only focus on the model accuracy; considering the parameter setting and mathematical meaning, as well as practicality, also matters. Therefore, the balance between model practicality and accuracy is crucial.

## 7. Conclusion and Future Work

The study compared the effectiveness of the LSTM and that of the Random Walk model in terms of financial issues. Moreover, the study explored the LSTM algorithm in combination with different parameter settings regarding different circumstances, respectively, the financial and medical issues. The main conclusion is that the LSTM model performs better than the Random Walk model. What needs to be noticed is the fact that the optimized parameters were surprisingly the same regarding financial and medical problems. Both were optimized at window length = 10 and prediction range = 5. In this regard, the optimal selection is suggested to explore in future work whether different circumstances have the same model parameters. As for the limitations, the range of window length settings is relatively large. Future research can be carried out within 10. Besides, the research objects are limited to Bitcoin and Ethereum, and more cryptocurrencies can be introduced for experimental modelling. Moreover, in the case of COVID-19, the data selection is only limited to mainland China which might not be representative, since the number of diagnosed cases is small. However, by comparing the model performance between financial and medical issues, the LSTM model parameter settings are suggestive in future work in a wide range of research pathways. Future work on the LSTM model application should focus on multiple combinations of the window length and prediction range parameters. It is advised to take the research results of window length = 10 and prediction range = 5 as a parameter setting cut-off to conduct comprehensive work in multiple research areas.

## 8. Additional Points

*Highlights.* The study uses different combinations of window sliding and prediction range settings to improve LSTM model. The study combines the Random Walk model and LSTM model based on economic theory to conduct experiment. By the insight of parameters settings in the financial case, the study applied the same parameters in the medical issues and verified the performance in both circumstances. The study proposes a view about the balance of model's accuracy and practicality based on the comparison of financial issues and medical issues. *Paraphrase.* Some of the ideas come from the author's master's dissertation in University of Southampton, which might be with some similarity in Turnitin. The supervisor agreed to use this idea as it is not a formal publishing.

## Figures and Tables

**Table 1:** The results of different model performance [12].

Model	Temporal length	Sensitivity (%)	Specificity	Precision	Accuracy (%)	RMSE (%)
LSTM	100	37	61.30%	35.50%	52.78	6.87
RNN	20	40.40	56.65%	39.08%	50.25	5.45
ARIMA	170	14.7	1	1	50.05	53.74

**Table 2:** Structure details of the article.

Section	Objective
Introduction	To clarify the research background and deep learning models as well as the article structure.
Literature review	To clarify the related work in the current topic.
Methodology	To introduce the mathematical principle of LSTM and Random Walk model.
Data collection	To introduce the data source.
Implementation	To train the models applied in financial and medical issues.
Discussion	To discuss the LSTM model performance and parameter settings in financial and medical cases.
Conclusion and future work	To give the final conclusion and future work suggestions.

**Table 3:** LSTM single-point prediction training process.

Epoch	18/20	19/20	20/20
step_loss	0.031	0.029	0.031
mean_absolute_error	0.031	0.029	0.031
val_loss	0.023	0.024	0.023
val_mean_absolute_error	0.023	0.024	0.023

**Table 4:** The error subtraction based on single day.

Window length = 10			
Days interval (based on single day)	4	9	14
Error subtraction, Bitcoin	0.005	0.013	0.025
Error subtraction, Ethereum	0.068	0.103	0.121

**Table 5:** The results of window length = 10 with different prediction range of COVID-19.

Window length = 10			
Prediction range	1	5	10
Loss error	0.0016	0.0015	0.0016

**Table 6:** The results of fixed prediction range with different window length.

Prediction range = 5			
Window length	10	50	90
LSTM MAE, Bitcoin	0.037	0.051	0.063
LSTM MAE, Ethereum	0.113	0.312	0.387

**Table 7:** The results of fixed window length with different prediction range.

Window length = 10				
Prediction range	1 (single point)	5	10	15
LSTM MAE, Bitcoin	0.032	0.037	0.045	0.057
LSTM MAE, Ethereum	0.042	0.113	0.145	0.163

## Data Availability

The data used to support the findings of this study are included within the article.
